# Fiducial-Aided Robust Positioning of Optical Freeform Surfaces

**DOI:** 10.3390/mi9020052

**Published:** 2018-01-30

**Authors:** Shixiang Wang, Chi Fai Cheung, Mingjun Ren, Mingyu Liu

**Affiliations:** 1 Parnter State Key Laboratory of Ultra-Precision Machining Technology, Department of Industrial and Systems Engineering, The Hong Kong Polytechnic University, Hung Hom, Kowloon, Hong Kong, China; Benny.Cheung@polyu.edu.hk (C.F.C.); renmj@sjtu.edu.cn (M.R.); samuel.liu@connect.polyu.hk (M.L.); 2 Robotics Institute, School of Mechanical Engineering, Shanghai Jiao Tong University, Shanghai 200240, China

**Keywords:** fiducials, positioning, freeform surface, precision metrology, robustness

## Abstract

Form characterization of a machined optical freeform surface demands accurate alignment of the sampled measured data points on the machined surface, and they are compared with the designed geometry of the surface through positioning. In this paper, a fiducial-aided robust positioning method (FAPM) is developed which attempts to evaluate freeform surfaces with high efficiency and precision. The FAPM method makes use of fiducials as reference datum to form a fiducial-aided computer-aided design (FA-CAD) of the freeform surface which not only establishes an inherent surface feature, but also links the different coordinate systems among design coordinate frame, machine tool, and measurement instrument. To verify the capability of the proposed method, a series of experiments were conducted. Compared with the traditional freeform measurement method (e.g., least squares method), the results indicate that the robustness and accuracy of the measurement is significantly enhanced by the FAPM.

## 1. Introduction

Freeform surfaces possessing non-rotational symmetry have been widely used in many industries, including aerospace, telecommunication, biomedical engineering, etc. [1]. This is because they can achieve excellent optical performance both in functionality and in size reduction [2]. To ensure the performance of the products, these surfaces are usually fabricated by advanced manufacturing technology such as being machined by three-to-five-axis computer numerical control (CNC) diamond cutting, milling, and polishing machine tools [3,4,5]. Because the machined surface is considerably influenced by its geometrical complexity, characterization of form accuracy of the machined surfaces is often carried out by comparing the extracted points (measured from the machined surface) with the design data. Since the two data sets are embedded in different coordinate systems, a slight misalignment between these two different coordinate frames can lead to large form errors during the assessment of the surface quality of the workpiece.

The unifying process of the two coordinate systems by a matching method is a non-linear optimization process based on approaches such as the least squares method (LSM) or the minimum zone method [6]. Many previous works have been studied, and this research has also achieved fruitful outcomes. Some modified iterative closest point (ICP) algorithms, which are based on intrinsic surface features such as mean and Gauss curvature [7,8,9] or local shape feature [10], were used to determine the optimal coordinate transformation parameters. However, these methods are considered to be easily trapped at local minima due to their small convergence domain. Moreover, the sensitivity of initial positioning (measured points in the measurement coordinate system) and lack of inherent features for flat surfaces pose relatively large challenges with the implementation of the ICP algorithms. Further, curvature-based localization methods are sensitive to the curvatures due to the machining errors [11], which means that the curvatures found on the machined surface may not be the same curvatures found on the designed surface because of the machining errors.

To improve the robustness and accuracy of positioning the machined surface in the measurement process, Kong et al. [12] proposed the coupled reference data method by machining additional surface features such as spherical and flat fiducials to evaluate flat optical surfaces. One obvious drawback of this method is that it needs to machine the references in the workpiece and the references need to be cut off if the machined surface is used in application. Youngworth et al. [13] used International Organization for Standardization (ISO) 10110 to show how to use the defined data and definition for metrology and data handing. A monolithic freeform element consisting of three spherical and one aspherical surface was successfully fabricated and fully evaluated using a machined reference datum (a vertex with three mutually perpendicular planes in the element). Although the defined references are sufficient for the measurement of surface angles, centration, and surface form deviation, these kinds of references probably cannot apply to all freeform surfaces because they do not serve as intrinsic surface features.

Zhang et al. [14] developed a positioning method based on fringe deflectometry, which can not only ensure the position of the freeform surfaces during machining, but also has high effectiveness in measurement data matching and optical alignment. However, the measuring devices need to be disassembled and assembled between the machining system and the measuring system with complex manual adjustments.

In this paper, a new method to preform high-precision positioning of freeform surfaces is presented. The method is named the fiducial-aided robust positioning method (FAPM) which makes use of fiducials as reference datum to generate a surface using fiducial-aided computer-aided design (FA-CAD), which not only establishes an additional surface feature but also links to different coordinate systems among the design coordinate frame, the machine tool, and the measurement instrument.

## 2. Fiducial-Aided Robustness Positioning Method (FAPM)

The proposed fiducial-aided robust positioning method (FAPM) was purposely developed to position the freeform surface among different coordinate systems of the designed surface model, the machine tools, and the measurement instrument. By using standard geometries such as spheres as basic element, some fiducials are designed and mounted on the fixture. These fiducials not only serve as intrinsic surface features, but are also used to estimate the parameters for the coordinate transformation.

**Figure 1:**
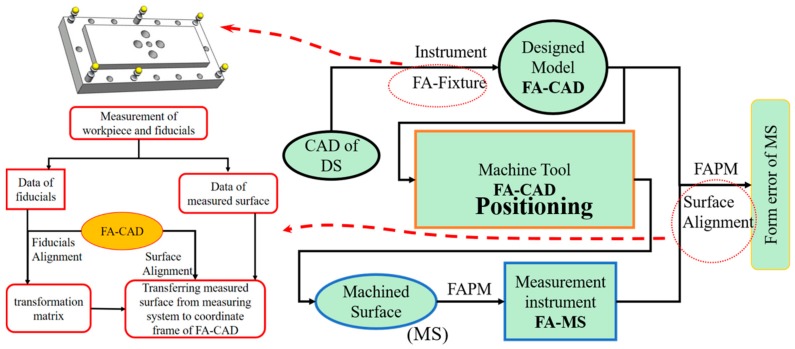
Schematic diagram of the fiducial-aided robust positioning method (FAPM). DS: designed surface; FA-CAD: fiducial-aided computer-aided design.

**Figure 2:**
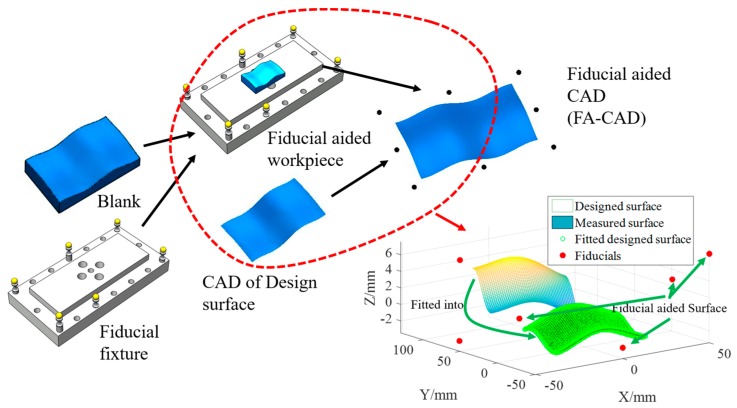
Construction process of the of FA-CAD of the designed surface (DS).

Figure 1 shows a schematic diagram of the FAPM. It starts with consideration of the CAD of the designed surface (DS), and a fiducial-aided fixture is purposely designed with the blank being assembled on it before the machining process. The fiducials (standard spheres) are designed with different heights so more information can be obtained to determine the effect of changes of the machining and measurement environment. Hence, a fiducial-aided computer aided design (FA-CAD) is generated by best fitting the CAD to the freeform surface in the fiducial-aided fixture (i.e., FA-fixture). It is clear that the fitted surface is the exact mathematical description of the designed surface. The process of the construction of the FA-CAD is shown in Figure 2. An on-machine measuring system is then incorporated into the machining system, which is used to measure the relative position of the fiducials. Hence, the fiducials serving as reference datum are used to determine a transformation matrix from the coordinate frame of the designed surface to that of the machining workpiece so as to transfer the FA-CAD to the coordinate frame of the machine tool. Finally, these fiducials are used to measure the machined surface on the off-machine measurement instrument as described in Figure 1. As a result, the fiducials provide intrinsic features of the FA-CAD to position the freeform surface both in the machining and the measurement processes.

It is interesting to note that the form characterization of the freeform surfaces—which are undertaken in the FAPM system—is free from the traditional least squares- or minimum zone-based best fitting processes. To reduce the deflection resulting from the thermal variation of the fixtures and the workpiece, the workpieces were measured in a thermally controlled environment (±0.2°).

To unify the reference coordinate frame (RCF) with the measuring instrument coordinate frame (MICF) and the machine tool coordinate frame (MTCF), fiducials were used to determine the homogeneous coordinate transformation matrix (HCTM). For example, to determine the position of the designed surface in the measurement instrument, it was assumed that there were *N* points in the reference coordinate system; the HCTM could be calculated by Equation (1) as follows:

(1) $$F\left(m\right)=\min\sum_{i=1}^{N}{\left|P_{i}^{MICF}-TP_{i}^{RCF}\right|}^{2},$$

where $P_{i}^{MICF}$ and $P_{i}^{RCF}$ are the central points of the ith fiducials in the RCF and the MICF, respectively. ***T*** is the homogeneous coordinate transformation matrix from the RCF to the MICF, and is defined by Equation (2):

(2) $$T\left(m\right)=\left[\begin{array}{cccc} c(r_{z})c(r_{y}) & s(r_{z})c(r_{y})+c(r_{z})s(r_{y})s(r_{x}) & s(r_{z})s(r_{x})-c(r_{z})s(r_{y})c(r_{x}) & t_{x}\\ -s(r_{z})c(r_{y}) & c(r_{z})c(r_{x})-s(r_{z})s(r_{y})s(r_{x}) & c(r_{z})s(r_{x})+s(r_{z})s(r_{y})c(r_{x}) & t_{y}\\ s(r_{y}) & -c(r_{y})s(r_{x}) & c(r_{y})c(r_{x}) & t_{z}\\ 0 & 0 & 0 & 1 \end{array}\right],$$

where *t_x_*, *t_y_*, and *t_z_* are the translation components, and *r_x_*, *r_y_*, and *r_z_* are the rotary angles, and are sorted in a vector ***m*** = (*r_x_*, *r_y_*, *r_z_*, *t_x_*, *t_y_*, *t_z_*); c() and s() are abbreviations of the cosine and sine functions. Then, Equation (3) defines the local minimum of Equation (1).

(3) $$\frac{\partial F}{\partial m}=2{\left(\frac{\partial R}{\partial m}\right)}^{T}R=0,$$

where ***R*** is the residual vector. Equation (3) is expanded with the Taylor series,

(4) $$\frac{\partial F}{\partial m}=2{\left(\frac{\partial R}{\partial m}\right)}^{T}R+2\left[{\left(\frac{\partial R}{\partial m}\right)}^{T}\frac{\partial R}{\partial m}+R^{T}\frac{\partial^{2}R}{\partial^{2}m}\right]\left(m^{*}-m\right)+\left[O{\left(m^{*}-m\right)}^{2}\right]=0.$$

By ignoring the higher-order terms in Equation (4), the Newton method [15] can be used to iteratively calculate the solution as follows:

(5) $$\delta m=-{\left(J^{T}J+S\right)}^{-1}J^{T}R,$$

where $J=\frac{\partial R}{\partial m}$ is a 3*N* × 6 Jacobian matrix; *N* is the number of fiducials. ***S*** is a 6 × 6 matrix with $S_{i,j}=R^{T}\frac{\partial^{2}R}{\partial m_{i}\partial m_{j}}$.

## 3. Experimental Investigation

To realize the performance of the FAPM, the method was implemented using MATLAB (The MathWorks, Natick, MA, USA) software and the program was run on an Intel Core i7 CPU 3.60 GHz PC with 16 GB of RAM. First, the robustness and accuracy (both rough and fine positioning) of the proposed FAPM were simulated on a complex surface (non-rotational symmetry freeform surface). A real freeform surface was then evaluated by the FAPM.

### 3.1. Simulation Study

A freeform surface was designed as described by Equation (6) as the designed surface (DS):

(6) $$\begin{array}{l} z=0.25(x+15)\cos(0.5x)+0.25(y+5)\cos(0.5y)+15\\ x\in [-15,15],y\in [-5,5] \end{array}.$$

**Figure 3:**
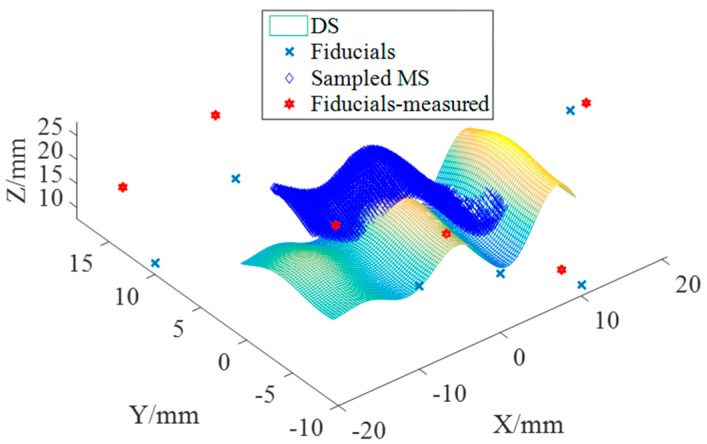
Simulated measured surface and designed surface with their fiducials.

There were six fiducials (standard spheres) that were designed surrounding the DS. The coordinate of the centers—which were calibrated—were (−20, 10, 10), (−10, 10, 20), (20, 0, 25), (10, −10, 10), (0, −10, 20), (−20, −10, 25) (unit is mm) in the reference coordinate frame (RCF). A number of points were uniformly sampled on the DS with spacing of 0.5 mm and 0.2 mm in the x and y directions, respectively. Hence, there were 3000 points sampled on the designed surface. To meet the practical operating environment, some uncertainties were considered to be added. These points were added machining errors (Gaussian distribution) with a standard deviation of 5 μm and measurement errors with a standard deviation of 0.7 μm. In addition, the error resulting from the determination of the centers of the sphere was considered as 0.3 μm. From the RCF, the DS and the fiducials were transformed to the measuring coordinate frame (MCF) with a known vector ***m***·(*R_x_*, *R_y_*, *R_z_*, *T_x_*, *T_y_*, *T_z_*) to represent the measured surface (MS). Figure 3 shows the fiducial-aided DS (FA-DS) and the fiducial aided MS (FA-MS). Both the least squares method (LSM) and the proposed FAPM were used to carry out the alignment process. Due to the complexity of the designed surface and the sensitivity for the initial value of the LSM, one set of good initial values with known vector ***m***_1_ (0.03, 0.07, 0.09, 0.2, 0.1, 0.1) were simulated as shown in Figure 3. The good initial value could be derived by transferring the sampled points to any known position so that the LSM could be easily carried out conversely.

The given spatial parameters were used to transform the calibrated fiducials and surfaces in the RCF to a new coordinate frame, which was considered as the MCF. Measurement errors and machining errors were added to the transformed fiducials and surfaces, respectively. In the LSM, the surface points were only used to search for the six spatial parameters of the coordinate transformation. In the FAPM, the six fiducials firstly served as intrinsic features to position the surface. So, the two datasets of the measured points and the designed points were unified in a common frame. Hence, the B-spline surface was used to represent the geometry of the datasets so as to evaluate the form errors. In order to find the accuracy of the proposed method, an additional LSM—namely fiducial-aided LSM (FA-LSM), where the fiducials were used to carry out the rough matching first before the LSM—was carried out to evaluate the six spatial parameters as well as the surface. Table 1 shows the evaluated errors of the six spatial parameters and the root-mean-square value (RMS) and peak-to-valley height (PV) under the ***m***_1_ vector. The definitions of RMS and PV height are shown in Equation (A1) in Appendix A. Δ denotes the difference of the six parameters resulting from the three methods and the ***m***_1_.

As shown in Table 1, these three positioning methods performed with good accuracy with relatively small deviation errors in terms of RMS and PV. Compared with the traditional LSM, there was an improvement (calculated as shown in Equation (A2) in Appendix A) of 31.4% and 32.2% in RSM, and about 14.5% in PV heights for the FAPM and FA-LSM respectively. Furthermore, the LSM converged after five iterations and the computation time was 1.26 s. Both the numbers of iterations and the time-consuming iterations underwent a significant decrease, down to 2 iterations (60%) and 0.197 s (81.9%) in the FA-LSM. In other words, it is clear that the FAPM and FA-LSM gave a higher accuracy of up to 1–3 μm compared to the traditional LSM.

To examine the robustness of the proposed method, the random vector was similar to ***m***_1_, in which only the last two translational parameters were slightly revised as given as ***m***_2_ (0.03, 0.07, 0.09, 0.2, 0.5, 0.5) was simulated.

It is shown in Table 2 that the obtained errors of the six parameters were very small (down to 0.083 × 10^−3^ rad for rotational transformation parameters and 1 μm translational transformation parameters) when employing the FAPM or FA-LSM, respectively. In contrast, the LSM converged to a local minimum with large errors, which was considered a failure to position the surface. As a result, the RMS and PV values were significantly larger than those achieved by the other two methods. The LSM consumed 1.23 s with six iterations, but FA-LSM only consumed 0.193 s with two iterations. According to the form errors, it seems that the FA-LSM could perform as well as the FAPM in terms of RMS and PV height values. However, taking the time-consuming iteration and the uncertainty resulting from the positioning process by using sampled points with machining errors into consideration, the FAPM was better than the FA-LSM. It is worth noting that the FAPM and FA-LSM match the measured surface to the most perfect position of the designed surface. In other words, the proposed FAPM makes use of fiducials to perform more robustly.

### 3.2. Experiments and Results

An optical freeform surface named an F-theta lens was defined by Equation (7) which was designed to verify the proposed FAPM:

(7) $$\begin{array}{l} z=(-1/250)x^{2}+(1/92000)x^{4}-(1/25)y^{2}\\ x\in [-18,18],y\in [-7.5,7.5] \end{array}.$$

**Figure 4:**
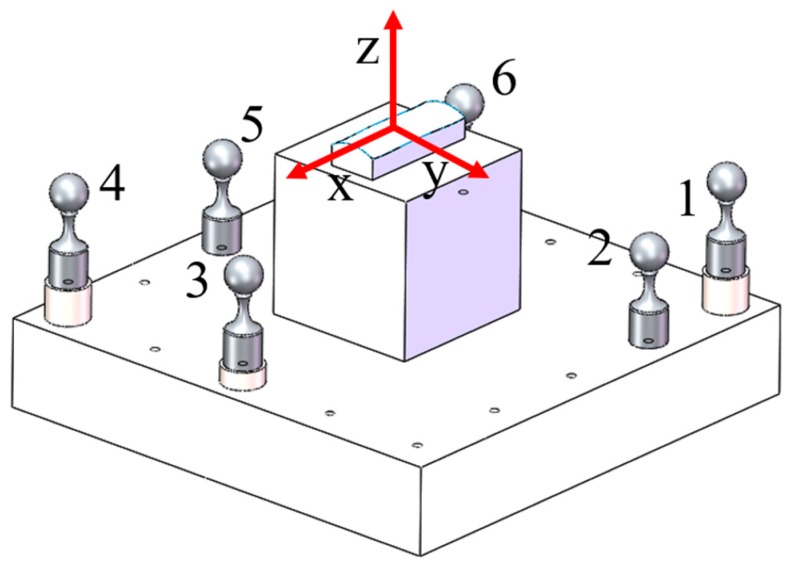
Designed fiducials fixture with a workpiece.

**Figure 5:**
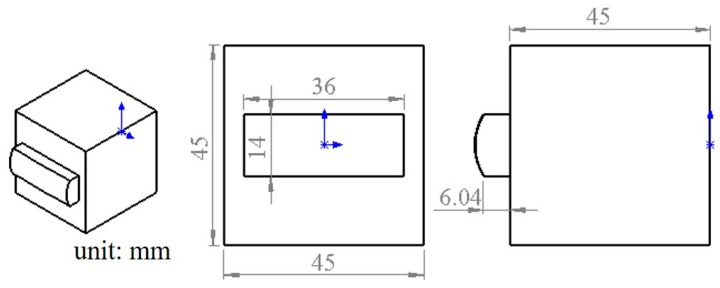
Dimensions of the designed surface.

A square fixture with dimensions of 140 mm (width) × 140 mm (length) was purposely designed as shown in Figure 4. The fixture was made of steel. The designated positions of the six calibrated spheres were used as the fiducials, which were located around the edges of the fixture, and they were designed with different heights. Taking the machine kinematics and tool collision into consideration, the center positions of the balls were designed as (−60, 60, −20), (−30, 60, −30), (60, 0, −25), (60, −60, −20), (0, −60, −30), and (−60, −30, −25) (unit is mm) in the reference coordinate frame (RCF). However, the mounted process inevitably introduced errors. The calibrated center positions of all the spherical fiducials which would serve as the RCF are summarized in Table 3. The calibrated diameter of the spherical fiducials bought from the market was 9.997 mm, and they were made of Si_3_N_4_. The workpiece was an aluminum cube with the dimensions of 45 mm × 45 mm × 50 mm and was fixed using screws. The dimensions of the surface and the workpiece are shown in Figure 5.

**Figure 6:**
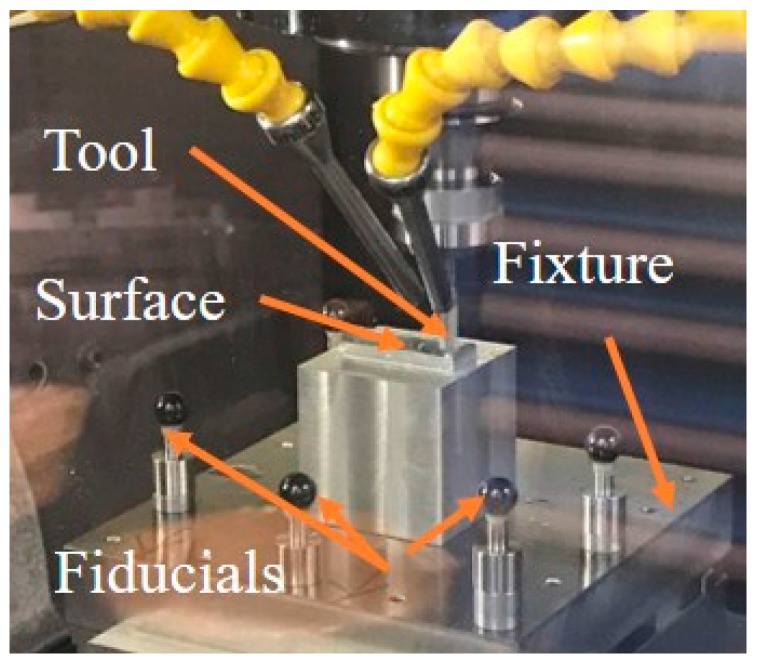
Machining surface by a machine tool.

**Figure 7:**
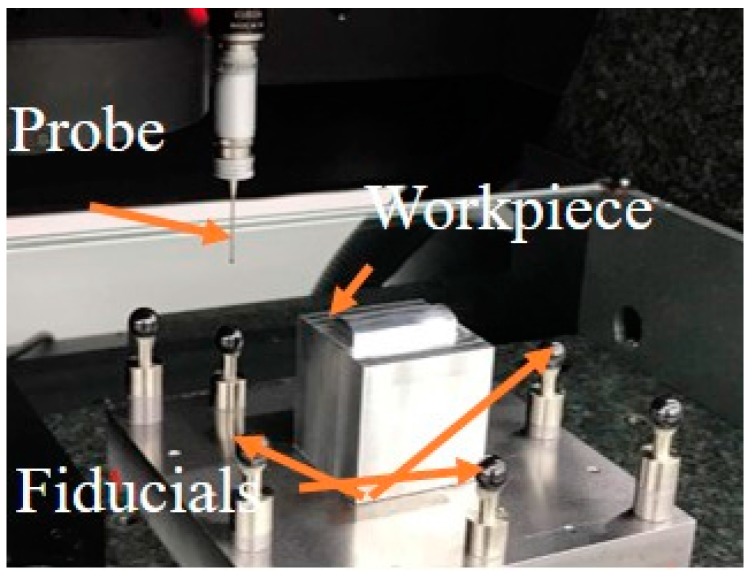
Measurement on a coordinate measuring machine (CMM) machine.

The designed surface was milled by a three-axis CNC machine (MIKRON UCP 600 Vario RTT, GF Machining Solutions, Geneva, Switzerland) with a ball-end milling cutter of 6 mm diameter, and the machined workpiece is shown in Figure 6. As shown in Figure 7, a multi-sensor coordinate measuring machine (CMM) machine (Werth Videocheck from Gießen, Germany) was used (a Renishaw trigger probe TP200, Renishaw, Hong Kong) to measure the freeform surface and calibrate the positions of the fiducials.

**Figure 8:**
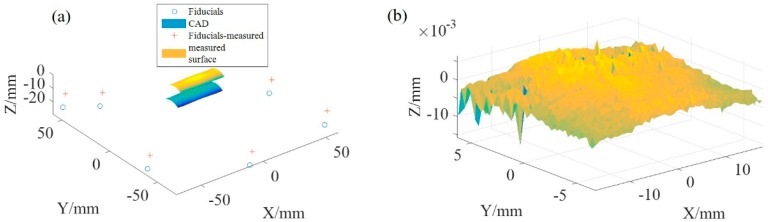
(**a**) FA-CAD and fiducial-aided measured surface (FA-MS); (**b**) 3D form error evaluation.

Figure 8a shows the 3D measurement data and the designed surface with their added fiducials and the matching result after positioning the measured points using the FAPM as shown in Figure 8b. According to the 3D form deviation, the evaluated surface error in terms of PV value was about 20.7 μm, while that for the RMS value was approximately 2.21 μm.

**Figure 9:**
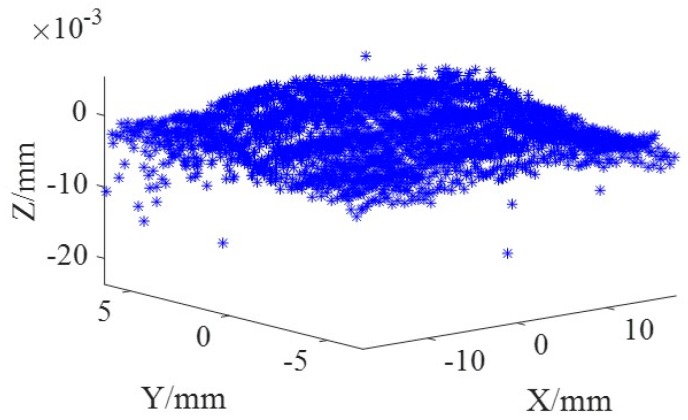
Point cloud of 3D error generated by LSM.

In addition, the LSM was also employed to obtain the form errors as shown in Figure 9. It was found that the PV value and RMS value were slightly larger than those resulting from the FAPM, with 29.2 μm and 2.5 μm, respectively.

### 3.3. Discusion

From the obtained PV height and RMS values by using the FAPM and LSM, it is clear that the FAPM achieved a better positioning accuracy. There was an improvement of 29% (from 29.2 μm to 20.7 μm) in PV height and 12% (from 2.5 μm to 2.21 μm) in RMS. The up to 8.5 μm difference (maximum deviation of the form errors between the FAPM and LSM) is considered to be the uncertainty caused by using the massive points with machined errors in the measured surface as well as the measuring uncertainties.

In this experimental study, the obtained form errors for the optical lens were significantly larger compared with the fabricated F-theta lens in [12] using ultra-precision raster milling technology with 0.64 μm and 5.66 μm in RMS and PV height, respectively; however, those results still prove that our proposed method can position freeform surfaces with wide availability because of the better results than for the LSM. Furthermore, the RSM and PV values obtained in the simulations had the same accuracy scale with RMS values of 2–3 μm and PV heights of 20–30 μm as those calculated in the experiment, which means that large form deviations probably resulted from the geometric errors, just like the standard deviation of 5 μm that was added to the measured surfaces in the simulation. In fact, the reason why a standard deviation of 5 μm was added was that the machine tool we used had almost the same machining accuracy.

According to the simulated and experimental results, it is interesting to note that both the RMS value and PV value were relatively smaller than the results obtained by the LSM. This is because the proposed FAPM makes the positioning process free from uncertainty caused by the measured points, which are usually massively sampled (usually several thousand points in an area measuring less than a few hundred mm^2^). Furthermore, once the FA-CAD is generated, the freeform surfaces can be positioned without the need to consider the shapes so that it can avoid the relatively time-consuming iterations.

## 4. Conclusions

In this paper, a robust positioning method has been developed to improve the performance in the positioning process to align the machined surface with the designed surface in different coordinate systems. A fiducial-aided CAD was integrated by using the fiducials and the designed surface to serve as a reference during the machining and measurement process. Both simulations and experimental study were carried out to demonstrate the effectiveness of our proposed method. The results can be summarized as follows:

1. The simulated results indicate that the proposed method had better matching accuracy (up to 32.2% in RMS and 14.5% in PV height) and robustness (lower uncertainty) than the traditional least squares method by using fiducials to link the coordinate systems between the machine tool and the measurement instrument, which means that the matching process is more robust to the errors introduced in the machining and measuring processes.
2. The results of the experiment show that the uncertainty (up to 8.5 μm) caused by a large number of iterations was minimized to improve the robustness for surface matching.

## Figures and Tables

**Table 1:** Evaluated errors of the six spatial parameters and the form accuracy.

*m*_1_		LSM	FAPM	FA-LSM
Spatial parameter errors	Δ*Rx* (rad) × 10^−3^	0.052	0.038	−0.027
	Δ*Ry* (rad) × 10^−3^	−0.024	0.008	0.004
	Δ*Rz* (rad) × 10^−3^	0.057	−0.003	0.081
	Δ*Tx* (mm) × 10^−3^	−0.443	−0.688	0.806
	Δ*Ty* (mm) × 10^−3^	−0.227	−0.363	0.584
	Δ*Tz* (mm) × 10^−3^	−0.171	0.097	−0.191
Form errors	RMS (mm) × 10^−3^	3.081	2.114	2.089
	PV (mm) × 10^−3^	23.664	20.219	20.242

LSM: least squares method; FAPM: fiducial-aided positioning method; FA-LSM: fiducial aided LSM. RMS: root-mean-square; PV: peak-to-valley height.

**Table 2:** Evaluated errors of the six spatial parameters and the form accuracy.

*m*_2_		LSM	FAPM	FA-LSM
Spatial parameter errors	Δ*Rx* (rad) × 10^−3^	−6.33	0.029	0.083
	Δ*Ry* (rad) × 10^−3^	5.93	0.016	0.018
	Δ*Rz* (rad) × 10^−3^	6.13	−0.016	−0.068
	Δ*Tx* (mm) × 10^−3^	7.13	−0.202	−0.031
	Δ*Ty* (mm) × 10^−3^	6.31	−0.456	−1.058
	Δ*Tz* (mm) × 10^−3^	4.86	−0.231	−0.226
Form errors	RMS (mm) × 10^−3^	16.689	2.121	2.119
	PV (mm) × 10^−3^	116.718	23.312	23.458

**Table 3:** Positions of the centers of the fiducials in the designed model.

Sphere	*X* (mm)	*Y* (mm)	*Z* (mm)
1	−59.79197	58.84895	−19.20588
2	−29.77590	59.14531	−29.00676
3	60.46800	−0.53976	−24.07613
4	59.47552	−59.93490	−19.65996
5	0.14544	−58.73153	−29.05214
6	−59.85573	−29.59255	−24.06871

## Appendix A

(A1) $$\begin{array}{l} \mathrm{RMS}=\sqrt{\frac{1}{N}\sum_{i=1}^{N}d_{i}^{2}}\\ PV=\max(d_{i}^{})-\min(d_{i}^{}) \end{array},$$

where $d_{i}=|P_{i}-Q_{i}|$, P*_i_* and Q*_i_* denote the data points of the measured points and the data points of the designed surface. *d_i_* denotes the distance between the design and measured surfaces.

(A2) $$\frac{{|V}_{FA-LSM/FAPM}{-V}_{LSM}|}{V_{LSM}}\times 100\%,$$

where V*_FA_*_–*LSM/FAPM*_ denotes the RMS or PV values or time-consuming iterations in the FA-LSM or FAPM, and V*_LSM_* denotes the RMS or PV values or time-consuming or iterations in the LSM.
